# Synthesis of Monoacryloxypropyl-POSS-based Hybrid Epoxyacrylate Copolymers and Their Application in Thermally Curable Structural Self-Adhesive Tapes

**DOI:** 10.3390/polym11122058

**Published:** 2019-12-11

**Authors:** Agnieszka Kowalczyk, Krzysztof Kowalczyk, Konrad Gziut

**Affiliations:** Department of Chemical Organic Technology and Polymeric Materials, Faculty of Chemical Technology and Engineering, West Pomeranian University of Technology in Szczecin, Pułaskiego 10, 70-322 Szczecin, Poland; kkowalczyk@zut.edu.pl (K.K.); konrad.gziut@gmail.com (K.G.)

**Keywords:** POSS, hybrid polymer, polymer blends, structural adhesives, pressure-sensitive tape, adhesion, mechanical properties

## Abstract

New organic-inorganic hybrid copolymers (EA-POSSs) based on butyl acrylate, glycidyl methacylate, hydroxybutyl acrylate, acryloiloxybenzophenone and acryloxypropyl-heptaisobutyl-POSS (A-POSS) were prepared via free-radical solution polymerization (FRP) and applied as a component of thermally curable structural self-adhesive tapes (SATs). The EA-POSS with 0.25, 0.5 or 1 mol % of A-POSS exhibited significantly higher dynamic viscosity (ca. +104%), *Mw* (+61%) and polydispersity (+109%; measured using gel permeation chromatography) as well as lower *T_g_* value (−16 °C) in relation to the A-POSS-free copolymer (EA-0). Differential scanning calorimetry (DSC) measurements (one glass transition process) confirmed statistic chain structure of the EA-POSS materials. Replacement of EA-0 by the EA-POSS copolymers in a SATs recipe caused simultaneous improvement of their self-adhesive features, i.e., adhesion (+70%), tack (+21%) and cohesion (+1590%). Moreover, the POSS-based copolymers improved the shear strength of thermally cured Al/SAT/Al overlap joints; the best mechanical resistance (before and after accelerated ageing tests) was observed for the sample containing 0.5 mol % of A-POSS (an increment range of 50–294% in relation to the A-POSS-free joints). Thermogravimetric analysis (TGA) revealed markedly improved thermal stability of the A-POSS-based SATs as well.

## 1. Introduction

Polyhedral oligomeric silsesquioxanes (POSSs) create a class of organosilicon materials with an empirical formula RSiO_3/2_ (where R is a hydrogen, alkyl, alkylene, aryl, arylene or other substituent). Depending on the functionality (mono- or multifunctional) and structure (random, ladder, cage or partial cage) of POSSs, they can be used as fillers, comonomers, cross-linking agents or initiators [1]. Incorporation of POSSs into polymeric materials can be realized by their blending (non-reactive POSSs or POSS-based polymers), as well as grafting and (co)polymerization (multi- and mono-functional POSSs) [2]. The first POSS-based copolymer was described by Lichtenhan in 1995 (a conventional free radical polymerization process of methacrylate-functionalized POSS monomers) [3]. Due to the unique features of POSS copolymers (e.g., high thermal stability), different POSS monomers were employed in atom transfer radical polymerization (ATRP), free radical polymerization (FRP), reversible addition fragmentation chain-transfer polymerization (RAFT), anionic polymerization processes, thiol-ene (click) and grafting reactions [4,5,6,7,8,9,10,11] or photoinitiated polymerization processes [12,13,14,15]. The first well-defined and high molecular weight polymers with POSS moieties were prepared by Marciniec in 2014 via the ATRP method [16]. Application of the POSSs in nanocomposites, light-emitting diodes, biomaterials and catalytic systems has been widely investigated [1,17]. Moreover, there are many publications describing POSS-modified organic coatings [18,19,20] and cast epoxy systems [21,22,23,24,25,26,27]. Generally, POSSs have an influence on the mechanical (higher modulus and elongation at break), thermal (higher *T_g_* and thermal stability, reduced thermal conductivity), barrier (better corrosion resistance) and other properties of polymeric materials [1,28]. It is noteworthy that copolymerizable POSSs can even contain eight reactive substituents (e.g., octamethacryloxy-POSS); thus, they are mostly used as crosslinking agents in thermal- or photo-initiated copolymerization/crosslinking processes of various (meth)acrylates. With the exception of [29,30,31,32,33], preparation of polymer networks based on octamethacryloxypropyl-POSS (known as 8M-POSS) and methyl methacrylate was presented in [34], while reactions of 8M-POSS with hydroxyethyl methacrylate, methyl methacrylate and dimethyl itaconate were investigated in [35]. Biomedical adhesives with monomethacryloxypropyl-POSS [36], structural epoxy adhesives with glycidyl-POSS [37] and cyanoacrylate dental adhesives modified with acrylo-POSS [38] are known; however, POSS-based adhesive materials have very rarely been considered in the literature. Moreover, there are no papers treating the application of monoacryloxyalkyl-POSS components in structural adhesives (including tape-type products) designed for the joining of metallic substrates. It is noteworthy that the first thermally curable structural adhesive (in a form of solid tape) was manufactured over 50 years ago by Hexcel from a phenolic resin and a polyvinyl formal; it was used for the production of the De Havilland 125 jet aircraft. Then, several adhesive tapes based on different phenolic and epoxy resins (additionally reinforced with polyester or polyamide fabrics) were developed [39]. A relatively new class of these materials is structural adhesive tapes with self-adhesive features (SATs). Generally, they can be prepared via UV-photocrosslinking process of reactive acrylate copolymers (compounded with an epoxy resin and a latent hardener) [40,41,42]. Unfortunately, due to the limited thickness of SATs, they do not contain reinforcing fabrics. Considering the above-mentioned literature (e.g., [1,28]), it seems that POSS is able to improve the mechanical and thermal properties of SAT-based joints. Thus, the aim of this work was to synthesize hybrid photosensitive epoxyacrylate copolymers (EA-POSS) based on acryloxypropyl-heptaisobutyl-POSS (A-POSS) and to compound thermally curable pressure-sensitive structural adhesive tapes (containing EA-POSS). The self-adhesive properties of the prepared SATs, as well as the mechanical and thermal features of thermally cured Al/SAT/Al overlap joints, were deeply investigated in this paper.

## 2. Materials and Methods 

### 2.1. Materials

**Figure 1:**
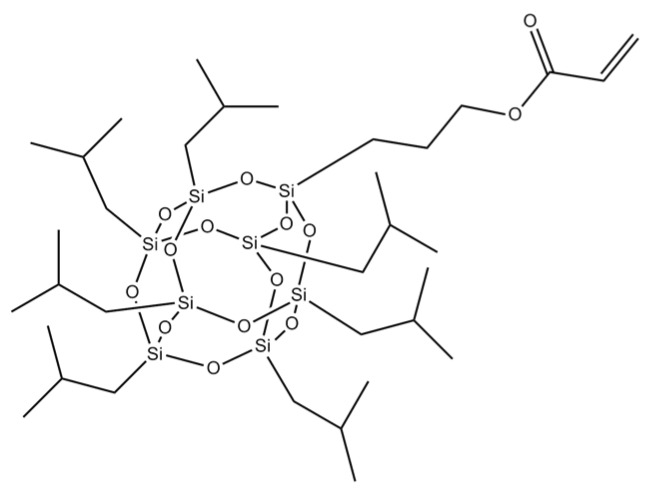
Structure of acryloxypropyl-heptaisobutyl-POSS (A-POSS).

**Figure 2:**
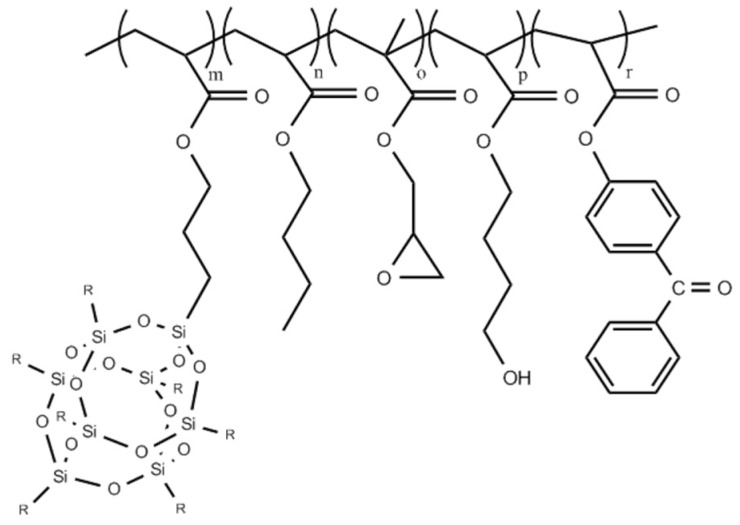
A theoretical structure of the EA-POSS copolymers.

The following components were used for preparation of hybrid epoxyacrylate copolymers (EA-POSSs): n-butyl acrylate (BA), glycidyl methacrylate (GMA), 4-hydroxybutyl acrylate (HBA) (BASF, Ludwigshafen, Germany), 4-acryloyloxy benzophenone (ABP; Chemitec, Scandiccy, Italy), acryloxypropyl-heptaisobutyl-POSS (A-POSS; Acryloisobutyl-POSS MA0701, C_34_H_72_O_14_Si_8_, *Mw*: 929.61 g/mol, Hybrid Plastics, USA, Figure 1), 2,2′-azobis(isobutyronitryle) (AIBN; Merc, Darmstadt, Germany) and ethyl acetate (POCh, Gliwice, Poland) as a solvent. The thermally curable double-sided structural self-adhesive tapes (SATs) were compounded using the EA-POSSs (or an A-POSS-free copolymer; EA-0), the Bisphenol A-based liquid epoxy resin with epoxy equivalent weight of ca. 202 g/eqiv. and viscosity 25 Pa·s (Epidian; Ciech Sarzyna, Nowa Sarzyna, Poland), the Lewis acid adduct (Nacure Super Catalyst A 218; Worleé Chemie, Hamburg, Germany) as a latent curing agent and modified poly(methylalkylsiloxane) (Byk 325; Byk-Chemie, Wesel, Germany) as a surface tension modifier.

### 2.2. Preparation and Characterization of the EA-POSS Hybrid Copolymers

**Figure 3:**
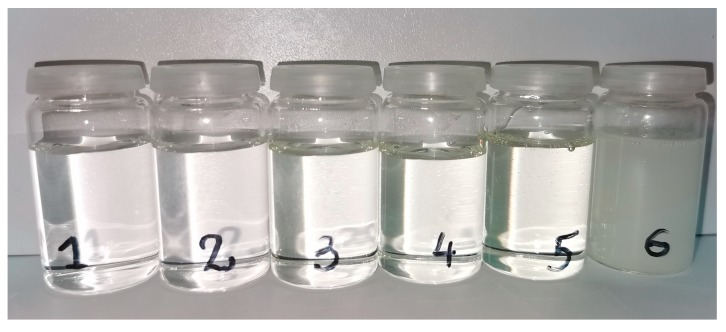
Solutions of the EA-POSS copolymers in ethyl acetate: EA-0 (**1**), EA-POSS-0.25 (**2**), EA-POSS-0.5 (**3**), EA-POSS-1 (**4**), EA-POSS-2.5 (**5**) and EA-POSS-5.0 (**6**).

The EA-0 and EA-POSS copolymers were synthesized via free radical batch copolymerization of A-POSS (0–0.5 mol), BA (7.5–8 moles), GMA (1 mol), HBA (1 mol) and ABP (0.001 mol) in ethyl acetate using AIBN as an initiator (0.1 wt part/100 wt parts of the monomers). The copolymerization process was realized at 78 °C for 5 h in a glass reactor equipped with a mechanical stirrer. The prepared products (i.e., copolymer solutions) contained 50 wt % of solids. Composition of the EA-0 and EA-POSS is tabulated in Table 1, while their theoretical structure is shown in Figure 2. A photograph of the copolymer solutions is presented in Figure 3.

Dynamic viscosity of the EA-0 and EA-POSS solutions was measured at 23 °C by means of the DV-II Pro Extra viscometer (spindle #7, 50 rpm; Brookfield, New York, NY, USA). Gel permeation chromatography (GPC-MALLS) was used for determination of molecular masses (*Mw*, *Mn*) and polydispersity (PDI) of the copolymers; the GPC apparatus contained the differential refractive index detector (Dn-2010 RI WGE Dr. Bures), the multiangle laser light scattering detector (DAWN EOS, Wyatt Technologies, Santa Barbara, CA, USA) and the columns: PSS 100 Å, PSS 500 Å, PSS 1000 Å and PSS 100000 Å (Polymer Standard Service, Mainz, Germany). The GPC tests were performed using THF and polystyrene standards.

### 2.3. Preparation and Characterization of Self-Adhesive Tapes (SATs) and Al/SAT/Al Joints

The SATs were compounded using the EA-0 or EA-POSS copolymers (50 wt parts), the epoxy resin (50 wt parts), the latent curing agent (1.5 wt parts), and the polymethylalkylsiloxane (0.75 wt part). The compositions were applied onto polyester foils (samples for self-adhesive tests) or siliconized paper (other tests), dried at 110 °C for 10 min and UV-irradiated for 40 s (15 J/cm^2^) using the medium pressure mercury lamp (UV-ABC; Hönle UV-Technology, Gräfelfing, Germany). The UV-exposition was controlled with the radiometer (Dynachem 500; Dynachem Corp., Westville, IL, USA). Base weight and thickness of the UV-photocrosslinked SAT layers were 150 g/m^2^ and 100 µm, respectively. Self-adhesive properties of the thermally uncured SATs were tested according to AFERA 4001 (adhesion to a steel substrate), AFERA 4015 (tack) and AFERA 4012 (cohesion). These parameters were evaluated using three samples of each adhesive tape. Differential scanning calorimetry (DSC Q100, TA Instr., New Castle, DE, USA) was used for determination of the glass transition temperature (*T_g_*) of the SATs, enthalpy of SAT curing processes (Δ*H*), onset temperature of the curing reactions (*T_i_*), and maximum temperature of the curing reactions (*T_p_*). Samples (ca. 10 mg) were analyzed using standard aluminum pans at the temperature range of −80–300 °C (heating rate of 10 °C/min). Two DSC measurements for each composition were carried out. 

Aluminum-SAT-aluminum overlap joints (Al/SAT/Al) were prepared using the SATs and degreased 2024 aluminum panels (100 × 25 × 1.6 mm); the joints were thermally cured at 170 °C for 60 min. Shear strength of the Al/SAT/Al systems was measured at room temperature according to the ASTM D1002-10 standard (ten samples of each system) using the Z010 machine (Zwick/Roell, Ulm, Germany). A long-lasting thermal ageing test was conducted acc. to MMM-A-132B (shear strength of the joints was measured after their storage at 82 °C for 192 h). Humidity resistance of the joints was determined (MMM-A-132B) after their 30-day exposure (40 °C, 95% RH) in the climatic chamber (LHU-114, Espec Corp.,Osaka, Japan). A fluid immersion test was realized acc. to MMM-A-132B as well; the overlap joints were immersed at 23 °C for 7 days in the aviation turbine fuel (Jet A-1; PKN Orlen, Warszawa, Poland).

Epoxy group conversion (EGC) in thermally cured SATs was analyzed using the FT-IR technique; variations of the absorbance value at 915 cm^−1^ (oxirane groups) were monitored and EGC values were calculated according to Equation (1) [43]:

(1) $$EGC={\left(1-\frac{A\left(t\right)}{A\left(0\right)}\right)}_{}\left(\mathrm{a.u.}\right)$$

where: *A*(0)—the initial intensity of the peak at 915 cm^−1^; *A*(*t*)—the intensity of the peak at 915 cm^−1^ after thermal curing of the sample. The mentioned peaks were normalized in relation to the reference peaks at 1182 cm^−1^ (C-O-Ar bonds of Bisphenol A-based diglycidyl ether [44]). Additionally, crosslinking degree (*α*) of the thermally cured SATs was calculated using DSC data according to Equation (2) [18]:

(2) $$\alpha ={\left(\frac{\Delta H_{T}-\Delta H_{res}}{\Delta H_{T}}\right)}_{}\left(\mathrm{a.u.}\right)$$

where: Δ*H_T_*—total enthalpy of a SAT curing process (J/g); Δ*H_res_*—enthalpy of a post-curing process of the thermally cured SAT (in a Al/SAT/Al joint). 

Thermal stability of the thermally cured SATs (in N_2_ atmosphere) was measured using the TG Libra analyzer (Netzsch, Selb, Germany). Samples (ca. 10 mg) were heated in aluminum pans at the temperature range of 25–800 °C (10 °C/min).

## 3. Results

### 3.1. Properties of A-POSS-Modified Epoxyacrylate Copolymers 

**Figure 4:**
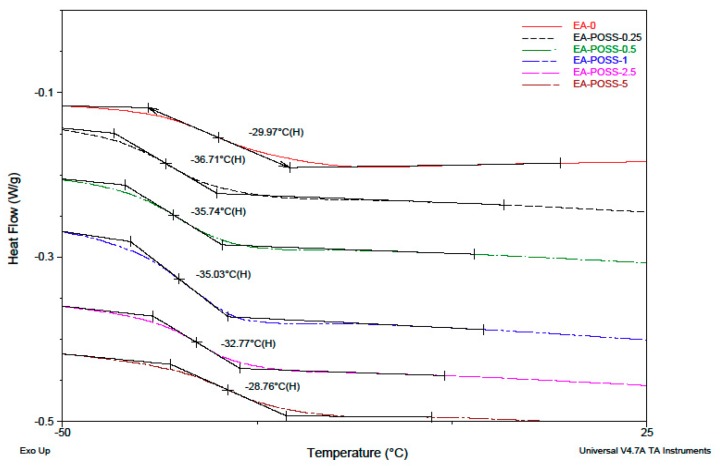
DSC thermographs for the epoxyacrylate copolymers modified with A-POSS.

Selected physico-chemical features (i.e., viscosity, molecular masses and glass transition temperature) of the epoxyacrylate copolymers (EA) chemically modified with monoacryloxypropyl-heptaisobutyl-POSS (A-POSS) are presented in Table 2. As can been seen, the hybrid copolymers prepared using the lower doses of A-POSS, i.e., 0.25 mol % (EA-POSS-0.25), 0.5 mol % (EA-POSS-0.5) or 1.0 mol % (EA-POSS-1), exhibited markedly higher viscosity (19, 25.9 and 15.6 Pa∙s, respectively) than the reference sample (EA-0, 12.7 Pa∙s). Interestingly, the viscosity of the samples with the higher A-POSS concentrations, i.e., 2.5 mol % (EA-POSS-2.5, 3.3 Pa∙s) and 5 mol % (EA-POSS-5, 1.5 Pa∙s), was dramatically reduced in relation to the other copolymers. It should be noted that the analyzed parameter values correlate with GPC measurement data. As can be seen in Table 2., the *Mw* of the modified copolymers generally decreases with increasing content of A-POSS; however, at the lowest modifier concentration (0.25 mol %), *Mw* reaches its highest value (ca. 703 kg/mol) in relation to the EA-0 and the other samples. Moreover, the PDI values (*Mw*/*Mn*) for EA-POSS-0.25 (6.63 a.u.), as well as for EA-POSS-0.5 (5.54 a.u.) and EA-POSS-1 (5.72 a.u.), were markedly higher than for the reference sample (3.22 a.u.) and samples with higher A-POSS content (3.24 a.u. for 2.5 mol %, 3.86 a.u. for 5 mol %). Generally, the GPC measurements indicate that the former group of the modified copolymers (with the lower A-POSS doses) does not have a unimodal molecular weight distribution (the high PDI). Nevertheless, all the EA-POSS samples exhibited only one *T_g_* value (i.e., the statistical structure of copolymer chains; Figure 4, Table 2), and the values of that parameters were lower than for EA-0 (−21 °C) and generally increased with increasing A-POSS dose (or decreasing butyl acrylate content; Table 1). It is noteworthy that the lowest *T_g_* was recorded for EA-POSS-0.25 (−37 °C), EA-POSS-0.5 (−36 °C) and EA-POSS-1 (−35 °C). This was probably a result of the higher *Mw* and PDI values of these copolymers in relation to the reference and other samples with A-POSS; a mixture of copolymer chains with markedly different length should generally be characterized by lower crystallinity and higher molecular mobility.

### 3.2. Properties of Thermally Uncured SATs with A-POSS-Based Epoxyacrylate Copolymers.

We have previously reported that the Bisphenol A-based epoxy resin (ER) and the epoxyacrylate copolymer (EA-0) are miscible components of SAT compositions due to the polarity of the two comonomers, i.e., glycidyl methacrylate and 4-hydroxybutyl acrylate. The ER/EA-0 mixture was characterized by one *T_g_* value revealed by the DSC technique [42]. In this research, we modified the epoxyacrylate copolymer with the organic-inorganic A-POSS comonomer; the resulting EA-POSS copolymers were used for preparation of thermally curable pressure-sensitive adhesive films (SATs). DSC data describing a thermal curing process of the SATs (based on the EA-POSS components) are presented in Table 3. As can be seen, only the SAT based on the EA-POSS-0.25 hybrid copolymer (i.e., SAT-POSS-0.25) exhibited one *T_g_* value (−18 °C), and this result was quite similar to *T_g_* of the reference SAT sample with the unmodified EA-0 copolymer (SAT-0, −19 °C). Thus, it can be claimed that ER is thermodynamically miscible with EA-0 and EA-POSS-0.25. In contrast, the other samples exhibited two *T_g_* values, indicating partial immiscibility of the epoxy resin and the epoxyacrylate copolymers prepared using 0.5–5 mol % of A-POSS. It should be noted that the first/lower *T_g_* decreased (while the second/higher *T_g_* increased) with increasing content of A-POSS; generally, the differences between the two recorded *T_g_* values were largest for SAT-POSS-2.5 (30 °C) and SAT-POSS-5 (28 °C). The observed phenomenon of the main reduction in *T_g_* values (from −19 °C for SAT-0 to −26 °C for SAT-POSS-5; Table 3) was affected by the increasing concentration of the POSS-type modifier in the systems. It is generally known that POSS molecules can cause a slip effect in polymer chains, resulting in a *T_g_* decrement [45]. Additionally, the ER/EA-POSS blends (based on the copolymers containing ≥0.5 mol % of A-POSS) were turbid—it confirmed that these ingredients are not totally miscible. Taking into consideration that the *T_g_* value for ER is ca. −10 °C [42], the second/higher *T_g_* value (Table 3) probably represents a glass transition process of a phase (consisting of a mixture of ER and selected chains of EA-POSS). In this case, it must be noted that the second *T_g_* values for the EA-POSS-based SATs generally increased with increasing *T_g_* value of the incorporated copolymers (Table 2). The second *T_g_* values may be affected by strong screening effects of POSS cages [46] embedded in the copolymer fraction miscible with the epoxy component. Nevertheless, the above-mentioned macroscopic incompatibility of ER and the majority of the EA-POSS copolymers (i.e., the main components of the SATs) did not negatively influence the mechanical properties of cured aluminum/SAT/aluminum joints (see Section 3.3). 

Generally, SATs based on EA copolymers with the lowest doses of A-POSS (SAT-POSS-0.25 and SAT-POSS-0.5) exhibited markedly lower enthalpy of cationic curing process of epoxy groups (Δ*H*) (185 J/g and 193 J/g), as well as higher onset temperature of the process (*T_i_*) (121 °C and 122 °C, respectively) in relation to the A-POSS-free SAT sample (SAT-0; 205 J/g and 112 °C; Table 3). Nevertheless, increasing content of A-POSS resulted in the increment of the Δ*H* parameter value (up to 215 J/g) and reduction of the *T_i_* value (107 °C for SAT-POSS-5). Interestingly, the mentioned relations are typical for SAT systems modified with fillers [41]. Arguably, relatively large POSS cages act as spherical hindrances and disturb the cationic polymerization of epoxy groups of the ER and EA-POSS components. The observed increment of exothermic peak temperature values (*T_p_*) for A-POSS-modified SAT samples confirm this thesis.

**Figure 5:**
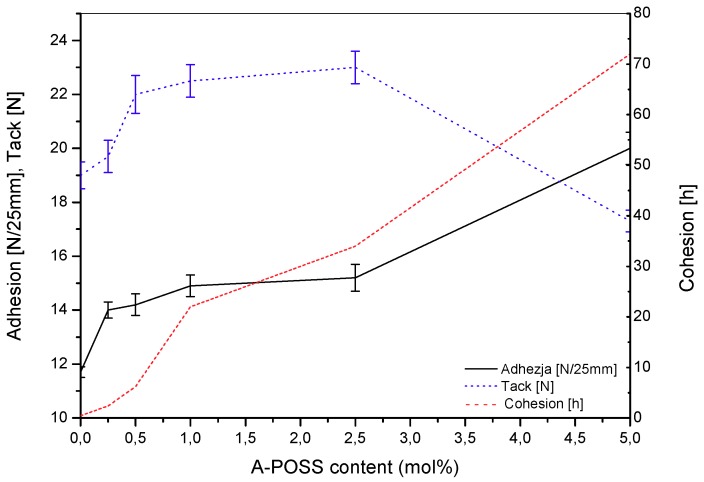
Adhesion to steel, tack and cohesion for thermally uncured SATs with A-POSS-based epoxyacrylate copolymers.

The influence of POSS structures on the adhesion of epoxy adhesives has not been widely described in the literature; moreover, there are no scientific papers concerning POSS-modified pressure-sensitive tapes and pressure-sensitive structural adhesives. Thus, the present research on SATs containing EA-POSS copolymers should be considered pioneering in this field. The basic self-adhesive features of the thermally uncured SATs are presented in Figure 5. The reference tape (SAT-0) reached relatively high adhesion (11.7 N/25 mm) and tack (19 N); however, its cohesion was low (0.45 h). In contrast, the SATs with EA-POSS-type copolymers exhibited significantly higher values of adhesion, cohesion and tack (the last parameter was improved at 0.5–2.5 mol % of A-POSS). Indeed, the lowest A-POSS dose (0.25 mol %, SAT-POSS-0.25) increased adhesion (14 N/25 mm, +20%) and cohesion (2.4 h, +433%); the higher A-POSS concentration resulted in further improvement of adhesion (+71%) and cohesion (72 h, ca. +150,000% for SAT-POSS-5). It should be noted that the high cohesion value (72 h) of classic pressure-sensitive adhesives can only be achieved with a highly effective cross-linking process and/or the high molecular weight of an applied acrylate copolymer (but adhesion is often reduced). In our investigations, all of the SAT compositions were UV-crosslinked thanks to the presence of 4-acryloyloxy benzophenone units in the EA copolymers. Interestingly, the incorporation of A-POSS cages into the EA copolymers (and SATs) simultaneously improved adhesion and cohesion. The former parameter probably increased due to two processes: (i) creation of hydrogen bonds between O atoms in the Si-O-Si structural elements of A-POSS cages and OH groups located on the surface of a steel panel used for the test (the higher A-POSS concentration, the higher number of the hydrogen bonds); and (ii) the A-POSS cages affect the UV-crosslinking process of SAT-POSS materials during the tapes preparation stage (due to the structural hindrances) and reduce crosslinking degree (*α*_uv_) of the adhesive films. It was previously reported that a decrement of *α*_uv_ could improve adhesion of pressure-sensitive tapes [47]. Arguably, the cohesion of the A-POSS-modified tapes increased due to their relatively higher stiffness observed during their preparation (despite the supposed reduction of *α*_uv_ values). In the case of tack, this parameter value increased with increasing A-POSS concentration up to 2.5 mol % (23.0 N, SAT-POSS-2.5); at higher modifier contents, it was reduced to 17.3 N (SAT-POSS-5). In relation to the tack measurement methodology, this parameter probably decreased as a result of the relatively high stiffness of the SAT-POSS-5 film as well. 

### 3.3. Properties of Thermally Cured SATs with A-POSS-Based Epoxyacrylate Copolymers

**Figure 6:**
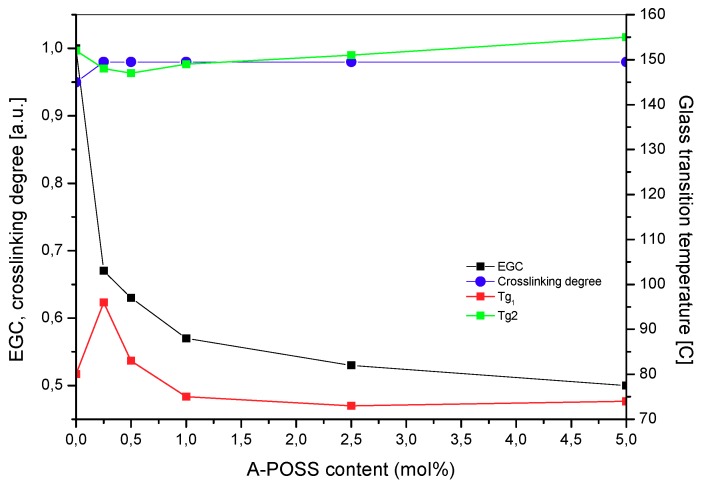
Epoxy group conversion (EGC, calculated using FTIR data), crosslinking degree and glass transition temperatures (calculated using DSC data) for thermally cured SATs with A-POSS-based epoxy copolymers.

The SATs containing A-POSS-based epoxyacrylate copolymers were applied onto 2024 T3 aluminum panels and the prepared Al/SAT/Al overlap joints were thermally cured at 170 °C for 60 min. As can be seen in Figure 6, the incorporation of A-POSS into the EA copolymer caused a reduction in epoxy groups conversion (EGC) calculated for thermally cured SATs using the FTIR method. EGC was markedly decreased from 1 a.u. (the peak at 915 cm^−1^ was not observed for SAT-0) to 0.5 a.u. for SAT-POSS-5. Arguably, a large size of A-POSS cages disturbed the curing process of epoxy groups via the creation of effective structural hindrances. Nevertheless, due to chemical incorporation of the cages into EA copolymer chains, this modifier did not cause such a dramatic decrement in the EGC parameter in comparison to the SATs modified with micro- and nanofillers [41]. On the other hand, crosslinking degree values (*α*; calculated using DSC data) for the thermally cured SATs (in all the Al/SAT/Al joints) were quite similar, i.e., 0.95 a.u. for SAT-0 and ca. 0.98 a.u. for the samples with A-POSS (Figure 6). Post-curing heat values recorded for the mentioned materials were similar, as well (ca. 3.5 J/g; data not presented). It is generally known that the DSC technique is a relative method (while the FTIR is an absolute method); thus, the *α* values (and EGC data) show that the thermally initiated curing process of epoxy groups was effectively blocked by the A-POSS presence in the EA copolymers. As a result, EGC should be taken into consideration as a more reliable parameter describing thermal crosslinking efficiency of SATs in the Al/SAT/Al joints. 

It has been presented in the literature that increasing the crosslinking density of thermosetting polymers (DSC data) results in a *T_g_* increment due to limitation of polymer chain segments mobility [48]. The realized DSC measurements of the thermally cured SATs revealed that these materials have two *T_g_* values (*T_g_*_1_ and *T_g_*_2_; Figure 6). This shows the presence of two polymeric (micro)phases or networks in the thermally cured samples, and is correlated with the DSC results for the uncured SATs (the two *T_g_* values; Table 3). The former *T_g_* values (recorded for the both types of samples) were probably related to the epoxyacrylate copolymer (without or with A-POSS), while the latter *T_g_* values represent the Bisphenol A-based epoxy component or phase. As can be seen, the cured SAT-POSS-0.25 exhibited higher *T_g_*_1_ (96 °C) and lower *T_g_*_2_ (148 °C; Figure 6) than the reference sample (SAT-0; 80 °C and 152 °C, respectively). Nevertheless, the higher doses of A-POSS caused a decrement of *T_g_*_1_ and an increment of *T_g_*_2_ (in relation to SAT-POSS-0.25). The above-mentioned initial limitation of *T_g_*_2_ was probably affected by the dramatic EGC reduction observed for the samples with the lowest A-POSS content. On the other hand, it is known that POSS molecules increase molecular rigidity of copolymers [23]; thus, the *T_g_*_2_ value increases at higher A-POSS concentrations. 

**Figure 7:**
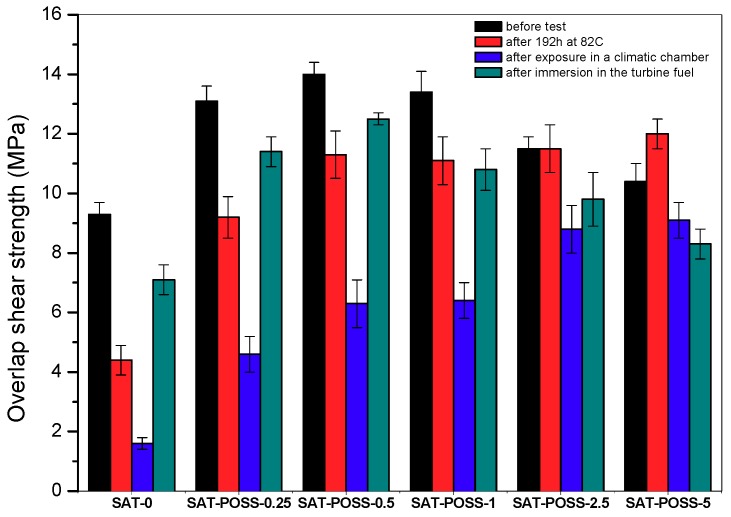
Shear strength for thermally cured Al/SAT/Al overlap joints (tested before and after different ageing tests).

Shear strength (*τ*) values recorded for the thermally cured Al/SAT/Al joints are presented in Figure 7. As can be observed, the reference samples (Al/SAT-0/Al) reached a lower value of the parameter (9.3 MPa) than those with A-POSS. Even the small dose of A-POSS (0.25 mol %) caused a significant improvement of the strength (13.1 MPa, +40%); however, the best result (14.0 MPa, +51%) was noted for Al/SAT-POSS-0.5/Al. A further increment of the modifier concentration resulted in slight reduction of *τ* to 13.4 MPa (+44%; the joints with SAT-POSS-1), 11.5 MPa (+24%, SAT-POSS-2.5), and 10.4 MPa (+12%, SAT-POSS-5). Generally, it is noteworthy that the highest *τ* values were registered for the samples containing the copolymers with the highest *Mw* (SAT-POSS-0.25, SAT-POSS-0.5 and SAT-POSS-1; Table 2). On the other hand, these SATs exhibited the considerable reduction of EGC in relation to the POSS-free system (Figure 6). A positive influence of lowered EGC on mechanical properties of SAT-based joints was revealed in [47]. The high *Mw* of the applied EA copolymer as well as the simultaneous low EGC values (i.e., a high content of unreacted polar epoxy groups in the thermally cured SATs) probably improved the shear strength of the tested joints.

Considering the *τ* of the cured Al/SAT/Al overlap joints (after different accelerated ageing tests; Figure 7), it can be claimed that the SAT-POSS-type samples exhibited markedly better thermal and chemical resistance than the reference joint. Generally, the mechanical strength of these joints (heated at 82 °C for 192 h) increased with increasing content of A-POSS; the Al/SAT-POSS-5/Al joints reached the highest *τ* value (12.0 MPa, +173%) in comparison to Al/SAT-0/Al (4.4 MPa) and other A-POSS-modified samples. Nevertheless, even the lowest dose of the cage-type modifier drastically improved the analyzed parameter of the thermally aged joints (Al/SAT-POSS-0.25/Al, 9.2 MPa, +109% vs. thermally aged Al/SAT-0/Al). The high thermal stability of POSS/epoxy systems (especially with epoxy-POSS) has been widely described in the literature (e.g., [49]). The resuts of thermogravimetric measurements (TGA) of the thermally cured SATs adhesives are presented in Table 4. As can be observed, the thermostability of the polymeric joints (represented by temperature value at 5% and 50% mass loss) was markedly increased by A-POSS incorporation into the EA copolymer structure. Interestingly, the highest *T*_5_ was recorded at the lowest A-POSS content (SAT-POSS-0.25, increment of 24 °C vs. SAT-0); in this case, *T*_50_ was also relatively higher in comparison to the reference samples (+32 °C) and the other A-POSS-based materials. It should be noted that SAT-POSS-0.25 was based on the copolymer exhibited the highest *Mw* (ca. 703 kg/mol, Table 2). Moreover, only EA-POSS-0.25 was visually miscible with the epoxy component; therefore, its dissipation in the SAT material should be better in comparison with the rest of A-POSS-based epoxyacrylate copolymers. These two aspects (i.e., the highest *Mw* and better compatibility with ER) probably resulted in the relatively highest thermal stability of the SAT-POSS-0.25 polymeric joint. On the other hand, a *Mw* value and A-POSS concentration arguably influence *T*_5_ and *T*_50_ for the materials with a higher content of the modifier (i.e., 0.5–5 mol %). It seems that increments of these parameters should improve thermal stability of the cured SATs. Therefore, the highest *T*_5_ and *T*_50_ values were simultaneously observed for the samples with the higher *Mw* (SAT-POSS-0.5; 338 and 434 °C, respectively) and with the higher A-POSS dose (SAT-POSS-5; 340 and 438 °C). In this case, the lowest thermal stability (except for the reference sample) was observed for the materials containing copolymers with the medium values of *Mw* and A-POSS content (SAT-POSS-1 and SAT-POSS-2.5).

Shear strength values for Al/SAT/Al joints (after their exposition in the climatic chamber) are presented in Figure 7. Generally, the recorded results correlate with those registered for the joints thermally aged at 82 °C for 192 h. After the 30-day humidity test at 40 °C (95% RH), the samples with A-POSS-based SATs reached markedly higher *τ* values than the A-POSS-free system (aged at the same conditions; 1.6 MPa); moreover, *τ* increased with increasing A-POSS concentration and the best mechanical resistance was noted for Al/SAT-POSS-2.5/Al (8.8 MPa, +450%) and Al/SAT-POSS-5/Al (9.1 MPa, +469% vs. aged joints with SAT-0). The A-POSS cages possibly limited migration of water vapor throughout the polymeric layer during storage of the joints at high humidity; the hydrophobic features of POSS were described in [20]. Interestingly, the A-POSS modifier efficiently improved chemical resistance of the joints to the aviation turbine fuel as well (Figure 7). It is noteworthy that the similar relation (between *τ* and A-POSS-content in the applied EA copolymers) was revealed for the non-aged Al/SAT/Al joints and the joints immersed for 7 days in fuel. In both cases, the highest shear strength values were observed for the samples based on SAT-POSS-0.5 (14.0 MPa and 12.5 MPa) and the percentage increment values (vs. non-aged and aged Al/SAT-0/Al samples) reached ca. 51% and 76%, respectively. The applied immersion medium probably did not act as a destruction agent of the Al/SAT/Al joints (e.g., water vapor), but as a plasticizer of the polymeric films. Thus, the shear strength of the reference and A-POSS-based overlap joints was uniformly reduced after their immersion test in the aviation fuel.

## 4. Conclusions

In this paper, the influence of acryloxypropyl-heptaisobutyl-POSS on selected features of epoxyacrylate copolymers (EA-POSS) was studied. Moreover, several physico-chemical, thermal and mechanical properties of thermally curable structural self-adhesive tapes with the copolymers (SATs), as well as Al/SAT/Al overlap joints were investigated. It was revealed that even a relatively small content of A-POSS (0.25, 0.5 or 1 mol % of the comonomers mixture) significantly affected dynamic viscosity, *Mw* and polydispersity, as well as the *T_g_* value of the copolymers. Considering the fact that DSC measurements revealed only one *T_g_* value for all of the copolymers, it can be claimed that EA-POSS have statistic chain structures [50]. Replacement of POSS-free epoxyacrylate copolymer by the EA-POSS in SAT compositions caused significant improvement of their self-adhesive features; shear strength of the thermally cured Al/SAT/Al joints was also increased. It seems that the properties of SATs and SAT-based joints mainly depend on the *Mw* and *T_g_* of the applied EA-POSS copolymers, as well as on epoxy group conversion in thermally cured SATs (containing the mixture of EA-POSS and Bisphenol A-type epoxy resin). In the case of the shear strength of Al/SAT/Al joints, the best mechanical resistance was generally observed for samples containing 0.5 mol % of A-POSS (i.e., Al/SAT-POSS-0.5/Al). Specifically, these joints exhibited higher or similar shear strength (before ageing, after thermal ageing or after the immersion test in the aviation turbine oil) in comparison to the reference sample and the other A-POSS-based systems. Higher resistance of the joints to water vapor (than Al/SAT-POSS-0.5/Al) was only recorded for the samples containing the highest concentration of the A-POSS modifier (2.5 or 5 mol %).

## Figures and Tables

**Table 1:** Composition of the epoxyacrylate copolymers with A-POSS.

Copolymer Symbol	Monomers (mol %)				
	A-POSS	BA	GMA	HBA	ABP
EA-0	0 (0 ^1^)	79.99	10.0	10.0	0.01
EA-POSS-0.25	0.25 (1.8)	79.74			
EA-POSS-0.5	0.5 (3.4)	79.49			
EA-POSS-1	1 (6.7)	78.99			
EA-POSS-2.5	2.5 (15.4)	77.49			
EA-POSS-5.0	5.0 (27.3)	74.99			

^1^ A-POSS weight concentration in an epoxyacrylate copolymer (wt %).

**Table 2:** Dynamic viscosity, molecular weight and glass transition temperature values for epoxyacrylate copolymers modified with A-POSS.

EA Acronym	*η* (Pa∙s)	*Mn* (g/mol)	*Mw* (g/mol)	PDI	*T_g_* (°C)
EA-0	12.7	135 500	436 800	3.22	−21
EA-POSS-0.25	19.0	105 900	702 700	6.63	−37
EA-POSS-0.5	25.9	114 700	635 800	5.54	−36
EA-POSS-1	15.6	105 600	604 600	5.72	−35
EA-POSS-2.5	3.3	128 100	415 400	3.24	−33
EA-POSS-5	1.5	85 900	331 700	3.86	−29

**Table 3:** Thermal features of uncured self-adhesive tapes (SATs) with A-POSS-based epoxyacrylate copolymers.

SAT Acronym	EA Type	*T_g_* (°C)	*T_i_* (°C)	*T_p_* (°C)	Δ*H* (J/g)
SAT-0	EA-0	−19	112	130/140/163	205
SAT-POSS-0.25	EA-POSS-0.25	−18	121	145/167	185
SAT-POSS-0.5	EA-POSS-0.5	−23, −12	122	143/176	193
SAT-POSS-1	EA-POSS-1	−25, −8	114	144/176	207
SAT-POSS-2.5	EA-POSS-2.5	−26, +4	110	145/173	212
SAT-POSS-5	EA-POSS-5	−26, +2	107	143/174	215

**Table 4:** Thermogravimetric analysis results for thermally cured SATs with A-POSS-based epoxy copolymers.

SAT acronym	*T*_5_^a^ (°C)	*T*_50_^b^ (°C)	Calcination residue at 700 °C (wt %)
SAT-0	321	404	0
SAT-POSS-0.25	345	436	0.7
SAT-POSS-0.5	338	434	1.0
SAT-POSS-1	329	433	4.1
SAT-POSS-2.5	331	427	5.3
SAT-POSS-5	340	438	9.7

a—temperature at 5% mass loss; b—temperature at 50% mass loss.
